# The Application of Seaweed Polysaccharides and Their Derived Products with Potential for the Treatment of Alzheimer’s Disease

**DOI:** 10.3390/md19020089

**Published:** 2021-02-04

**Authors:** Sarah Bauer, Weihua Jin, Fuming Zhang, Robert J. Linhardt

**Affiliations:** 1 Center for Biotechnology and Interdisciplinary Studies, Department of Chemistry and Chemical Biology, Rensselaer Polytechnic Institute, Troy, NY 12180, USA; bauers2@rpi.edu (S.B.); jinweihua@zjut.edu.cn (W.J.); 2 College of Biotechnology and Bioengineering, Zhejiang University of Technology, Hangzhou 310014, China; 3 Center for Biotechnology and Interdisciplinary Studies, Department of Chemical and Biological Engineering, Rensselaer Polytechnic Institute, Troy, NY 12180, USA; zhangf2@rpi.edu; 4 Center for Biotechnology and Interdisciplinary Studies, Departments of Biological Science, Biomedical Engineering, Rensselaer Polytechnic Institute, Troy, NY 12180, USA

**Keywords:** Alzheimer’s disease, polysaccharides, seaweeds, neuroprotective activity

## Abstract

Neurodegenerative diseases are among the most widespread diseases affecting humans, and the number of patients is only rising. Seaweed polysaccharide extracts show significant neuroprotective and reparative activities. Seaweed polysaccharides might provide the next big breakthrough in neurodegenerative disease treatment. This paper reviews the applications of seaweed polysaccharides as potential treatments of neurodegenerative diseases. The particular focus is on fucoidan, ulvan, and their derivatives as potential agents to treat Alzheimer’s disease. This review provides a critical update on the progress in this important research area.

## 1. Introduction

Neurodegenerative diseases are characterized by the progressive loss of cognitive and physical function. The most common neurodegenerative diseases are Alzheimer’s disease (AD), Parkinson’s disease, Huntington’s disease, and Amyotrophic Lateral Sclerosis (ALS) [1]. There are two main types of neurodegenerative diseases: movement disorders and degeneration/dementia disorders. AD is the most common degenerative disorder [2].

AD is an irreversible progressive neurodegenerative disease leading to memory loss and cognitive deficit [3]. The etiology of AD is not fully understood, as many factors impact disease progression and presentation [4]. Alois Alzheimer first discovered this disease in 1907, which was when the two main characteristics of Alzheimer’s were first discussed, namely, amyloid-beta plague and neurofibrillary tangles [5]. There are several contributing factors to AD development and progression, including apoptosis, oxidative stress, neuroinflammation, mitochondrial dysfunction, cholinic dysfunction, and abnormal protein development [6]. An excess of D-galactose can be an early sign of Alzheimer’s disease progression. This sugar conjugates with glucose to form lactose, and when there is too much D-galactose vs. the amount of glucose present, metabolic dysfunction and oxidative stress occur [7].

As medical science progresses the average human lifespan increases, leading to a rising number of AD cases [8]. Almost 6 million people exhibited AD symptoms in 2018; of these cases, 200,000 were early onset, occurring in people less than 65 years old [9]. The number of AD patients is projected to increase to over 100 million by 2050 [10]. The cases of Alzheimer’s disease in the world have been doubling every 20 years, with projections for 2040 at around 80 million [11].

Alzheimer’s is known as a tauopathy, meaning that in Alzheimer’s patients there is a high concentration of misfolded and insoluble tau protein. The first location where this insoluble protein builds up is in the hippocampus [12]. This is what forms the trademark neurofibrillary tangles found in Alzheimer’s patients. This misfolding of protein is common in all neurodegenerative diseases as these proteins control brain function. The concentration of tau in the human body is determined by two systems: the autophagy-lysosome pathway and the ubiquitin-proteasome pathway. When autophagy is suppressed in mice models of Alzheimer’s, neurodegeneration quickly follows indicating that failure in this pathway can contribute to the neurodegeneration found in Alzheimer’s. There is also a high concentration of unubiquitinated tau found in models where the autophagy pathway has been compromised, indicating these systems may be linked. When these systems function properly, the tau protein is soluble and moves throughout the brain and body causing no damage, but when they fail, tau becomes insoluble due to misfolding and can build up due to the blood–brain barrier preventing the release of such insoluble particles. Amyloid-beta is another protein commonly found in the human body; it is usually soluble and passes through the body without causing harm. Amyloid-beta becomes neurotoxic when it aggregates and misfolds, the most toxic species being amyloid-beta_42_ [5]. The true nature of what makes hyperphosphorylated tau and aggregated amyloid-beta neurotoxic is still not understood and requires more research. These two misfolded proteins are the ideal targets for potential Alzheimer’s disease treatments [5].

**Figure 1:**
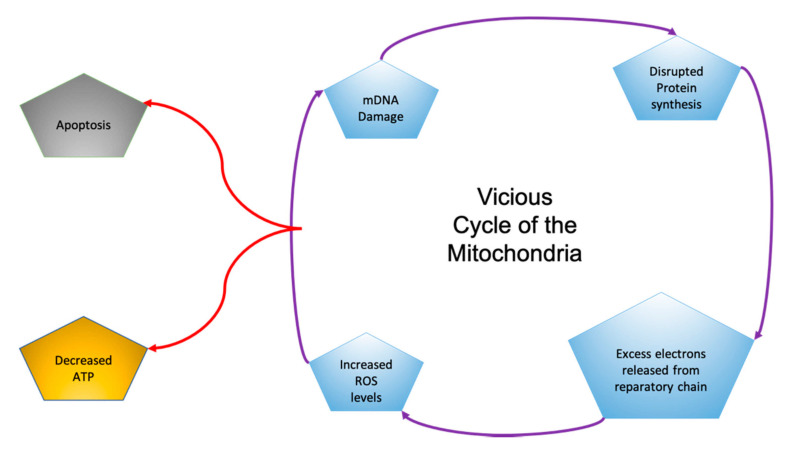
In the “Vicious Cycle of the Mitochondria”, each aspect of damage leads to subsequent dysfunction and affects other aspects of the cycle [9].

Mitochondria are energy-producing organelles that control cell survival and neuronal cell death [13]. Mitochondria produce the energy molecule ATP, with reactive oxygen species as byproducts. When mitochondrial DNA is mutated, the mitochondria can produce excess reactive oxygen species, leading to oxidative stress. This starts a vicious cycle in the mitochondria in which mDNA mutates, leading to the overproduction of ROS, resulting in oxidative stress and DNA mutations (Figure 1) [9]. Brain tissue is very sensitive to oxidative stress due to its high concentration of unsaturated fatty acids and the inability of the brain to regulate itself like other organs [13].

Approximately 90% of the Earth’s biomass is found in the ocean, and marine organisms represent about half of the world’s known species [14]. Marine macroalgae, or seaweed, make up a large portion of this biomass with over 10,000 species found globally [15]. Due to the ready availability of seaweed globally, it has been used in medicines for millennia, ever since the year 3000 BC [9].

Seaweed is classed into three categories based on pigmentation: green seaweed (*Chlorophyta*)*,* red seaweed (*Rhodophyta*), and brown seaweed (*Phaeophyta*). There are over 4000 species of red seaweed, over 900 species of green seaweed, and over 1500 species of brown seaweed worldwide. Brown seaweed can be found in temperate water, while red and green seaweeds grow exclusively in tropical waters [4].

Polysaccharides serve as energy reserves and as structural components and are found in all organisms. Many types of seaweed contain over 80 wt.% polysaccharides [16]. Seaweeds frequently rely on sulfated polysaccharides as cell wall material to aid in their flexibility to prevent tidal damage [17]. Sulfated polysaccharides, such as those found in seaweed, have been shown to exhibit high anti-inflammatory and antioxidant activities (Table 1) as well as the ability to scavenge free radicals [16]. Oxidative stress and inflammation are some of the leading agents in the progression of AD [3]. Nutraceuticals protect against the formation of reactive oxygen species and subsequent tissue damage; thus, seaweed polysaccharides may be a good source of nutraceuticals [18].

Sulfation has been shown to directly impact the bioactivity of polysaccharides. In ROS-compromised cells, cell viability increased by 40% when treated with sulfated polysaccharides and only by 10% when treated with the same non-sulfated polysaccharides. This is probably due to the increases in free radical scavenging associated with sulfated polysaccharides [7].

Unfractionated heparin was used as a reference for the study shown in Table 2, which has an IC_50_ of 0.29 ± 0.02 μg/mL [19]. The most sulfated sample A09-SP had a degree of sulfation (DS) value of 0.81 and had a lower IC_50_ value than the reference sample. The increase in sulfation showed a significant improvement in IC_50_ value between fraction 2 and fraction 3. This demonstrates that increasing sulfation drastically lowers the IC_50_ [19].

## 2. Application of Polysaccharides from Brown Algae in Treating AD

### 2.1. Phaeophyta

**Figure 2:**
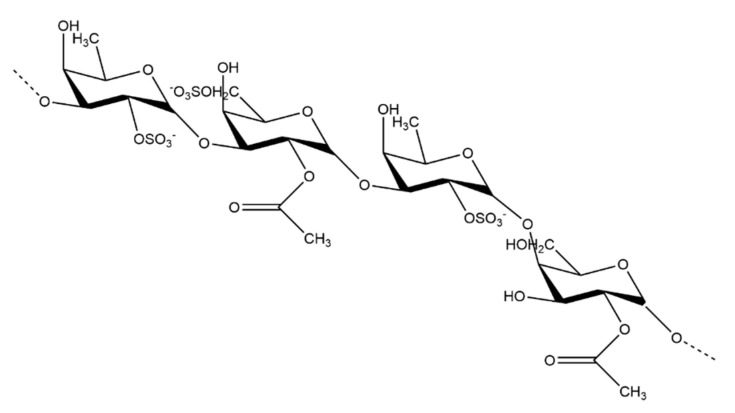
Generalized structure of the fucoidan polysaccharide found in brown seaweed.

The sulfated polysaccharide found in brown seaweed is fucoidan. There are many variations in fucoidan structure depending on the species of seaweed, but their overall general structures are quite similar. Fucoidans (Figure 2) primarily contain sulfated L-fucose residues [22]. Some species of brown seaweed also contain a compound known as lamaran. Lamaran consists of a β-(1-3)-glucan with β-(1-6)-linkages of 20–25 units [27]. Another main polysaccharide found in brown seaweed is alginate, found in the cell wall of some seaweed species [28].

### 2.2. Findings of Recent Studies

An in vitro study found that polysaccharide extracts from *Ecklonia radiata* (a brown seaweed) prevented apoptosis and amyloid-beta toxicity, and revived compromised cells after oxidative damage [10]. Fucoidans have been found to inhibit ROS production [22], as well as to inhibit the formation of nitric oxide (NO) and prostaglandins [29]. In a 2018 study, polysaccharide extracts from *Sargassum muticum* (a brown seaweed native to Japan) were added to 6-OHDA comprised cells. Because it increases hydrogen peroxide concentration leading to oxidative stress and decreased cell viability, 6-OHDA has been used to study neurodegenerative damage. All known fucoidan extracts scavenge DPPH, ABTS, and FRAB used in monitoring toxic free radicals [2]. *Ecklonia cava* polysaccharide extracts also lowered mitochondrial-mediated protein expression and protein aggregation [13].

**Figure 3:**
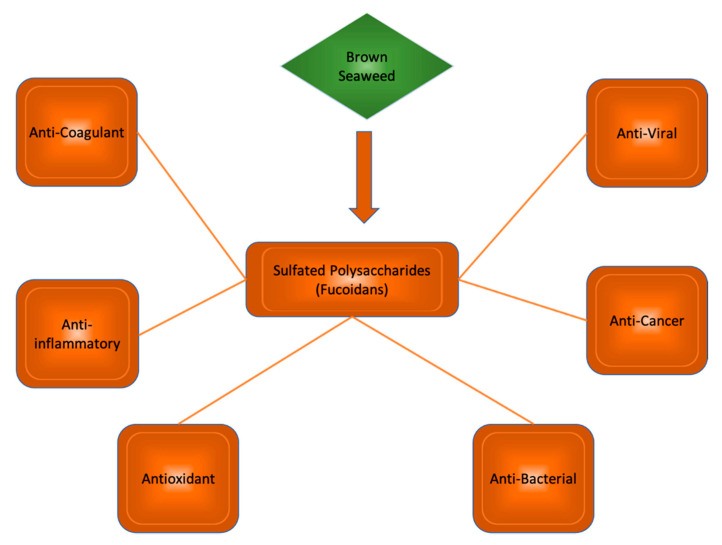
Sulfated polysaccharides in brown seaweed species and their bioactive properties.

Polysaccharides from brown seaweeds show many bioactive properties (Figure 3). These properties include anti-inflammatory, antioxidant, anticholinic, and regulatory activities. Studies suggest their potential use in the treatment of neurodegenerative disease. Mice exhibiting signs of neurodegeneration showed improved memory and learning when treated with fucoidan extracts [14], suggesting great promise for similar sulfated polysaccharides in future human trials.

Another recent study details a trial in which neurologically compromised mice were fed polysaccharide extracts from the brown seaweed *Sargassum fusiforme* [20]. LXRβ and LXRα are liver X receptors (LXRs) in the brain. The activation of LXRβ improves cognition and reduces amyloid-beta plaques in AD patients; however, activation of LXRα can lead to hypertriglyceridemia and hepatic steatosis, making this a difficult treatment route [20]. Phytosterols found in plants/foods common to Western diets do not activate LXRs. A series of plants used in Eastern diets were tested for their in vitro ability to activate LXRs. *Sargassum fusiforme* extracts showed the best activation of LXRβ, with limited activation of LXRα, and resulted in cell death at 5 µg/mL. This concentration was then tested on AD mice, with a control group not receiving treatment. The mice treated with *Sargassum fusiforme* polysaccharide extracts showed a significant reduction in amyloid-beta plaques, improved memory, and improved cognition, as compared to both the baseline and the control group [20]. A series of tests were performed on the cardiovascular systems of mice using alginate oligosaccharides. The tests all showed that the pretreatment with alginate oligosaccharides vastly improved the amount of myocardial infarction and cell apoptosis. This study also looked at the ability of a pretreatment with active oxygen species (AOS) to reduce oxidative stress in mice tissue. The generation of ROS and the expression of protein were measured after reperfusion and myocardial infarction, events that would normally drastically increase the levels of ROS, especially the NO, present in cardiac cells. Pretreatment with AOS not only mitigated this reaction but lowered the overall ROS content in the cardiac cells. This suggests a similar reaction is possible in brain tissues suffering oxidative stress [30].

## 3. Application of Polysaccharides from Green Algae in Treating AD

### 3.1. Chlorophyta

Green seaweed, known as Chlorophyta, has slightly fewer polysaccharides per dry weight than brown seaweed, with 77% of its mass being polysaccharides; these polysaccharides contain 21% sulfate [31]. The primary sulfated polysaccharide found in green seaweed is ulvan. The sulfate content of each polysaccharide has been directly correlated to its antioxidant and neuroprotective potential.

### 3.2. Findings of Recent Studies

Green seaweed has also been investigated for its nutraceutical potential. A study was conducted to test the in vivo as well as the in vitro protective effects of sulfated polysaccharides from green seaweed [32]. In hydrogen peroxide-compromised cells, the cell viability and amount of apoptosis both improved after treatment with polysaccharide extracts from green seaweed. Zebrafish were treated with high levels of hydrogen peroxide to lead to oxidative stress in order to test the neuroreparative effects in animals. The survival rate for the fish treated in this way was very low; when the second group of zebrafish was treated with hydrogen peroxide and green seaweed polysaccharide extracts, the survival rate increased due to a decreased level of ROS. This led to a recovery in some of the zebrafish, demonstrating the potential for neurorepair in animals [32].

## 4. Application of Polysaccharides from Red Algae in Treating AD

### 4.1. Rhodophyta

Red seaweeds (Rhodophyta) provide many bioactive constituents, such as proteins, polysaccharides, pigments, polyunsaturated fatty acids, and phenolic compounds. Polysaccharides account for 40–50% of their dry weight. Agar and carrageenan are the two main types of cell wall polysaccharides from red seaweed, and are important in nutritional, medical, and industrial products [33].

### 4.2. Findings Findings of Recent Studies 

Sulfated polysaccharides from the red alga *Gelidium pristoides* incubated with Aβ1–42 showed disappearance of Aβ1–42 fibrils, suggesting the activity of disaggregation and inhibition of aggregation of the fibrils [26]. к-carrageenan oligosaccharides were reported to exhibit immunomodulatory function by acting on LPS-activated microglial cells, resulting in their biological activity for preventing inflammation-related neurodegenerative diseases [34]. Another study showed that к-carrageenan-derived pentasaccharide can attenuate Aβ 25–35-induced apoptosis through the JNK pathway [35]. The scavenging activity of the polysaccharide carrageenan was determined. IC_50_ values of 36.6 and 32.8 mg/mL were measured for naturally extracted carrageenan and commercially available carrageenan, respectively [36]. This suggests a similar scavenging activity for carrageenans from different sources [35].

## 5. The Mechanism of Polysaccharides from Seaweeds for the Treatment of AD

Fucoidan has been shown to increase GPX levels and prevent ROS production [37]. High levels of acetylcholinesterase cause cholinergic dysfunction, which leads to memory loss in AD patients. Seaweed polysaccharide extracts inhibit both acetylcholinesterase and butyl cholinesterase, and this could improve cholinergic deficit in patients [31]. Fucoidan extracts inhibit the production of ROS, inhibit the aggregation of amyloid-beta leading to toxicity, and significantly improve hydrogen peroxide toxicity in mice models. Apoptosis due to oxidative damage is also decreased [10]. The body’s natural defense against oxidative stress is antioxidants; sulfated polysaccharides from brown seaweed show more antioxidant activity than any other known compound, perhaps due to their high content of sulfation [38]. Seaweed polysaccharides reduce ROS production and scavenge O_2_^−^ and OH, leading to increased cell viability and decreased apoptosis, and improving memory and learning function [39]. By increasing SOD1 and SOD2, oxidative stress can be avoided or reduced in cells treated with seaweed polysaccharide extracts [40]. Lamaran has also been found to inhibit the production of hydrogen peroxide, the most damaging ROS [29].

Neuroinflammation is a controlling factor for both damage and repair of brain tissue [37]. Inflammatory responses are present around amyloid-beta plaques in AD [41]. Seaweed polysaccharides decreased lipid peroxidation and erythrocyte hemolysis, leading to decreased inflammation of cells [39].

BACE-1 is a protease that regulates the production of amyloid-beta, and fucoidan extracts inhibit BACE-1, leading to decreased production of amyloid-beta. By limiting the aggregation of amyloid-beta, seaweed polysaccharide extracts decrease the cytotoxicity of amyloid-beta [31]. Fucoidan can ameliorate spatial learning or memory defects by preventing the formation of amyloid-beta plaques [37].

**Figure 4:**
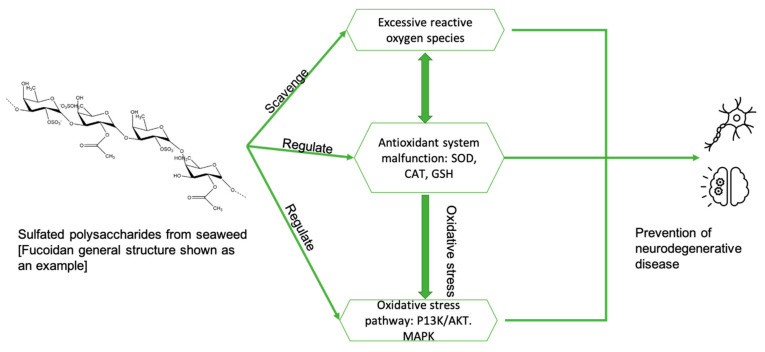
The possible mechanisms of sulfated polysaccharides in regard to neurodegenerative disease progression. Adapted from [22].

In summary (Figure 4), the possible mechanisms for the treatment of Alzheimer’s disease using seaweed extracts (poly/oligo saccharides) include: (i) anti-inflammatory and antioxidant activities; (ii) scavenging free radicals; (iii) inhibition of ROS production and inhibition of the formation of nitric oxide (NO) and prostaglandins; (iv) lowering mitochondrial-mediated protein expression and protein aggregation; (v) directly interacting with the aggregated peptide, preventing oligomerization and fibrillation of Aβ; (vi) attenuation of Aβ-induced apoptosis through the JNK pathway; and (vii) impacting gut microbial processing and subsequent neuroinflammation.

## 6. Challenges and Opportunities

**Figure 5:**
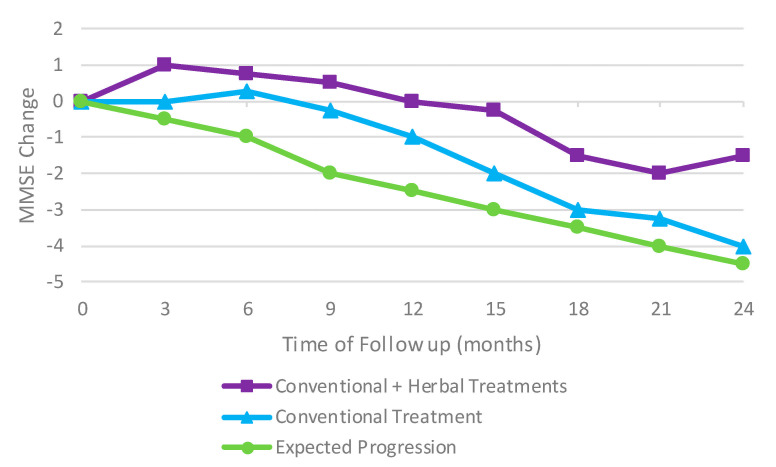
An improvement in mini-mental state examination (MMSE) scores compared to the initial baseline and the expected untreated progression of AD. Results adapted from [42].

China has a long history of using herbal medicine to treat a variety of illnesses including dementia. In 2017, a retroactive cohort study was conducted to see if the addition of herbal medicine to conventional treatment options for AD could improve the cognitive ability of the patients or even slow the progression of the deterioration. Patients showed an improvement in their mini-mental state examination (MMSE) scores compared to the initial baseline and the expected untreated progression of AD (Figure 5). The normal decline in MMSE scores was significantly delayed when treated with both conventional treatments and herbal treatments [42].

Seaweed is not the only source of natural compounds being explored as an option in the treatment of AD. A Korean group looked at the impact that long-term treatment with red ginseng extracts could have on Alzheimer’s patients. This study was 24 weeks long, with 61 patients undergoing treatment. During the 24-week trial, the patients’ AD assessment scale score improved markedly when compared with the control group. More important than the initial 12-week and 24-week scores were the scores that were seen over the next two years: these scores showed no deterioration once treatment ceased [11,43].

Some species of seaweed have a large quantity of non-sulfated polysaccharides along with their sulfated polysaccharides; this is true for other medicinal plants as well. Non-sulfated polysaccharides that had undergone chemical sulfation, in place of their naturally sulfated counterparts, were also shown to reduce oxidative stress [7]. This addition of sulfate groups onto polysaccharides extracted from *Rhodiola sachalinensis* using chlorosulfonic acid-pyridine suggests the same practice could be applied to other seaweed polysaccharides to increase their level of sulfation.

Seaweed is currently widely available globally but because of climate change, the availability of seaweed could change. A study was conducted to see if polysaccharides from seaweed grown in bacterial hosts could be sulfated, affording sulfated polysaccharides with the same bioactivity as naturally occurring sulfated polysaccharides [44]. This study found that in cells pretreated with the synthetic sulfated polysaccharides, the level of hydrogen peroxide required to induce oxidative stress was decreased. The survival rate of cells treated with only hydrogen peroxide was less than 60%, while the cells that were pretreated with 0.5 µg/mL sulfated polysaccharides showed a survival rate of nearly 100%. The highest concentration, 2.0 µg/mL, was the least effective concentration to ameliorate the oxidative damage; this concentration still resulted in a cell survival rate of nearly 90%. This demonstrates that chemical modification is a useful method for mass-producing sulfated polysaccharides that could provide a potential solution to oxidative stress injury and subsequent apoptosis in neuronal cells [44].

One of the main challenges in AD treatment is a therapeutic’s ability to navigate the blood–brain barrier. One solution to overcome the blood–brain barrier may be to attach these molecules to nanoparticles, relying on nanotechnology to overcome this obstacle [45]. A nanotechnological approach demonstrated that curcumin attached to nanoparticles could cross the blood–brain barrier. Curcumin has also been proposed as a possible treatment for AD due to its ability to bind to amyloid-beta, preventing aggregation [46]. This study found that a relatively low dose of nanoparticles (23 mg/week) when delivered across the blood–brain barrier showed improvements in memory and cognition in mice with AD symptoms [46]. A similar study using seaweed polysaccharides immobilized to nanoparticles could represent a new approach for patients suffering from AD.

Currently, the only treatment options for AD are cholinesterase inhibitory medications. These medications cause liver damage and do not block AD progression and, in some cases, they even exacerbate this progression [47]. Current research suggests that polysaccharide extracts from brown seaweed show low cytotoxicity, relatively high bioavailability, and low production costs and, thus, might offer alternative therapeutics [48]. These polysaccharides may represent ideal candidates for drug research and discovery. As of this publication, there have been no positive results in human trials to confirm the utility of seaweed polysaccharides in treating AD [45]. As with any possible treatment, the difficulties in undertaking human trials still represent a large barrier to success.

China has particularly high rates of AD, with nearly one third of the population over 90 being affected. China has issued conditional approval for a new AD drug, GV-971, to address this growing problem [49]. GV-971 stems from a study performed to test the impact of sodium oligomannate on gut microbial processing and subsequent neuroinflammation [50]. The 5XFAD transgenic mouse model, a standard mouse model commonly used in AD studies, mimics amyloid-beta formation as well as cognitive deficits. After one month of receiving oral GV-971, the affected mice showed a different gut microbiome as well as improved cognition [50].

This breakthrough suggests the possible use of seaweed polysaccharide extracts in treatment options for AD. Testing the efficacy of seaweed polysaccharides will require careful monitoring and data tracking to confirm the utility of such treatments in humans.

In conclusion, polysaccharides from seaweed could very well be an effective treatment for AD and other neurodegenerative diseases. The current data are very positive and show great potential, but additional research is required to establish efficacy, low toxicity, and beneficial human responses, as well as to examine other possible interactions that could undermine the neuroprotective capability of these polysaccharides. Additional studies on a wider variety of seaweeds, as well as human efficacy trials, are required. Finally, additional studies on the mechanism of seaweed polysaccharides in relation to AD symptoms and progression are needed to further the understanding of the effects of these polysaccharides.

## Figures and Tables

**Table 1:** A compilation of seaweed species used in recent Alzheimer’s disease (AD) research.

Seaweed Species	Classification	References	Activity Presented in References
*Saccharina latissima*	Brown	[19]	Anti-Inflammatory
*Sargassum fusiforme*	Brown	[20,21]	Antioxidant, Antiamyloidogenic
*Ecklonia cava*	Brown	[22]	Antioxidant
*Sargassum fluitans*	Brown	[22]	Antioxidant
*Turbinaria decurrens*	Brown	[22]	Antioxidant
*Laminaria japonica*	Brown	[23]	Neuroreparative, Antioxidant
*Undaria pinnatifida*	Brown	[22]	Antioxidant
*Caulerpa lentillifera*	Green	[24]	Antioxidant
*Ulva lactuca*	Green	[25]	Antioxidant
*Gelidium pristoides*	Red	[26]	Antioxidant, Antiamyloidogenic

**Table 2:** A comparison of sulfation content and IC_50_. Adapted from [19].

Polysaccharide Sample	Degree of Sulfation (DS)		IC_50_ Elastase (μg/mL)	
Fraction	F2	F3	F2	F3
B06-SP	0.28	0.82	1.87 ± 0.12	0.26 ± 0.02
A05-SP	0.22	0.76	2.81 ± 0.21	0.28 ± 0.01
A09-SP	0.19	0.81	3.77 ± 0.16	0.21 ± 0.01
